# Assessment and applications of joint profiling of single-cell chromatin accessibility and transcriptome

**DOI:** 10.1093/bib/bbaf669

**Published:** 2025-12-12

**Authors:** Hongfei Li, Jiechen Wang, Qing Liu, Quan Zou, Ximei Luo

**Affiliations:** Yangtze Delta Region Institute (Quzhou), University of Electronic Science and Technology of China, No. 1 Chengdian Road, Kecheng District, Quzhou 324003, China; Institute of Fundamental and Frontier Sciences, University of Electronic Science and Technology of China, No. 2006, Xiyuan Ave, West Hi-Tech Zone, Chengdu 610054, China; Yangtze Delta Region Institute (Quzhou), University of Electronic Science and Technology of China, No. 1 Chengdian Road, Kecheng District, Quzhou 324003, China; Institute of Fundamental and Frontier Sciences, University of Electronic Science and Technology of China, No. 2006, Xiyuan Ave, West Hi-Tech Zone, Chengdu 610054, China; Department of Anesthesiology, Hospital (T.C.M) Affiliated to Southwest Medical University, No. 319, Section 3, Zhongshan Road, Luzhou 646099, China; Yangtze Delta Region Institute (Quzhou), University of Electronic Science and Technology of China, No. 1 Chengdian Road, Kecheng District, Quzhou 324003, China; Institute of Fundamental and Frontier Sciences, University of Electronic Science and Technology of China, No. 2006, Xiyuan Ave, West Hi-Tech Zone, Chengdu 610054, China; Yangtze Delta Region Institute (Quzhou), University of Electronic Science and Technology of China, No. 1 Chengdian Road, Kecheng District, Quzhou 324003, China; Institute of Fundamental and Frontier Sciences, University of Electronic Science and Technology of China, No. 2006, Xiyuan Ave, West Hi-Tech Zone, Chengdu 610054, China

**Keywords:** single-cell sequencing, transcriptomics, joint profiling, chromatin accessibility, *cis*-regulatory elements, bioinformatics tools

## Abstract

Joint profiling technologies combining single-cell chromatin accessibility (CA) and transcriptome sequencing enable cellular heterogeneity analysis from both gene and *cis*-regulatory element perspectives, greatly advancing molecular biology at a cellular resolution. These techniques have been used to construct gene regulatory networks across diverse cell types and biological tissues, contributing significantly to the mapping of cell developmental trajectories. In this review, we summarize existing single-cell joint profiling methods for CA and transcriptomics and systematically evaluate the data quality of each modality using consistent criteria: the median number of genes detected per cell (RNA) and the median number of accessible peaks per cell (ATAC). Furthermore, we examine relevant bioinformatics tools and highlight their applications in various omics research contexts. Finally, we discuss the current limitations of joint profiling technologies, prospects for future improvement, the extensibility of computational tools, and the potential for co-assaying with additional omics data.

## Introduction

The biochemical functions of proteins underpin the diversity and autonomy of cells, tissues, and organisms [1–3]. Regulatory proteins, known as transcription factors (TFs), synergistically interact with *cis*-regulatory elements (CREs) to control gene transcriptional levels and drive cell fate decisions, facilitating the establishment of unique expression profiles in different cell types [4–7]. For example, overexpression of TFs such as NEUROD1, FERD3, and LMX1B induces the differentiation of human embryonic stem cells into peripheral neurons, whereas overexpression of FLI promotes their differentiation into vascular endothelial cells [8]. Signal proteins act as cellular messengers, mediating intercellular communication via ligand–receptor interactions and activating downstream pathways that determine cell differentiation trajectories [9–12]. For instance, binding of the BMP receptor (BMPR2) on mesenchymal stem cells to the BMP2 ligand activates the Smad signalling pathway, triggering osteogenesis-related gene expression (GE) and driving osteogenic cell differentiation [13]. Immunoglobulins serve as a critical line of defence against foreign pathogens in mammals by enhancing host immunity and neutralizing pathogen activity through antigen binding [14–16]. For example, during SARS-CoV-2 infection, immunoglobulins bind to the viral spike protein, restricting viral entry, thus informing therapeutic strategies for COVID-19-associated acute respiratory syndrome [17]. While protein diversity supports essential biological processes, protein synthesis ultimately depends on gene transcription and translation, which are regulated by the binding and modification of genomic elements. Therefore, understanding the regulatory mechanisms of protein-coding genes is fundamental for mapping gene regulatory networks in cells, tissues, and organisms.

The rapid advancement of sequencing technologies has provided robust technical support for quantifying transcriptional products, CREs, and epigenetic modifications in tissue samples [18–21]. Transcriptome sequencing enables measurement of GE levels, identification of key gene sets, and functional characterization of biological samples. Epigenomic sequencing uncovers chromatin architecture and epigenetic modification patterns, offering insights into regulatory networks and developmental processes. Integrating multiple omics layers paves the way for biomarker discovery, reconstruction of gene regulatory network, and the development of novel disease treatment strategies [22, 23]. However, bulk sequencing fails to resolve cell-specific regulatory mechanisms in complex tissues like the tumour microenvironment. Single-cell sequencing overcomes this limitation by capturing omics signals at cellular resolution [24, 25]. In single-cell RNA sequencing (scRNA-seq), RNA is converted into complementary DNA (cDNA) using reverse transcriptase, which is then amplified and sequenced, allowing GE quantification at single-cell resolution. scRNA-seq has been used to identify key cell populations in various diseases and detect disease-specific marker genes [26–28]. In a single-cell assay for transposase-accessible chromatin using sequencing (scATAC-seq), Tn5 transposase is used to label accessible chromatin regions, which are then amplified and sequenced based on adapter sequences [29, 30]. This technology has been used to identify numerous CREs specific to disease-related cells and tissues in different species, providing essential tools for elucidating pathogenic mechanisms and modelling cellular differentiation trajectories.

Single-cell sequencing technology facilitates the annotation of specific cell types and the identification of CREs [31–35]. However, as cellular development and disease mechanisms are inherently multidimensional, the integration of multiple omics modalities is essential for their comprehensive understanding [36, 37]. Emerging joint-profiling technologies enable simultaneous measurement of chromatin accessibility (CA) and GE within the same cell [38, 39]. These approaches, combined with expanding datasets, have advanced the mapping of differential regulatory programs, reconstruction of developmental trajectories [37, 40], elucidation of cell fate decisions [41, 42], and multi-perspective characterization of disease-related cell heterogeneity [43, 44]. Considering that elucidating gene regulatory networks is a central goal of the Human Cell Atlas [45–48], this review summarizes current progress in joint profiling of single-cell CA and transcriptomes, highlights recent technological innovations, and discusses the challenges and opportunities for decoding cellular regulation.

## Joint profiling of single-cell CA and transcriptome

**Figure 1:**
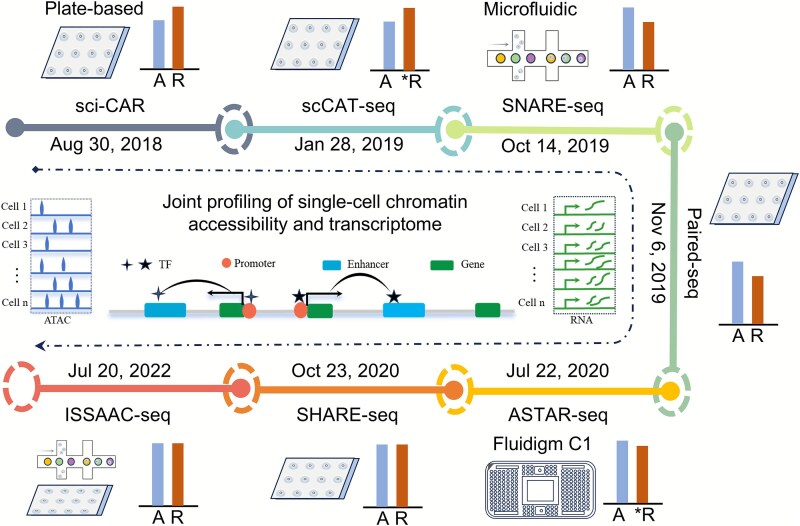
Developmental timeline of techniques for joint profiling of single-cell CA and transcriptomes. The bar chart represents data quality. R: RNA (transcriptome), A: ATAC (accessibility), ^*^R: full-length transcript and low throughput.

Single-cell CA profiling provides insights into the potential cell fate decisions by examining genomic activity, specifically through the identification of CREs, while single-cell transcriptome profiling determines cell developmental trajectories and biological functions by measuring gene transcription levels. However, there is limited understanding of how cells with identical genomic coding information within the same individual give rise to diverse cellular phenotypes. To address this, researchers have developed joint profiling techniques, which can simultaneously measure single-cell CA and transcriptomes (Fig. 1). These approaches aim to deconstruct the dynamic regulatory relationships between chromatin states and GE, providing a multidimensional and multi-perspective framework for characterizing cell type maps, tracing cell lineage trajectories, and dissecting disease mechanisms.

### sci-CAR

In 2018, Cao *et al.* developed the sci-CAR protocol, which integrates sci-ATAC-seq and sci-RNA-seq through a two-round indexing strategy [49]. In the first round, barcodes and unique molecular identifiers (UMIs) were assigned to RNA from isolated nuclei via reverse transcription (RT) using a poly(dT) primer, while barcodes were assigned to ATAC fragments through *in situ* tagmentation using the Tn5 transposase. In the second round, RNA-derived cDNA and ATAC fragments were amplified, further barcoded at the single-cell level, and then sequenced (RNA-seq and ATAC-seq, respectively). sci-CAR was used for CA and transcriptome profiling of dexamethasone-treated A549 lung cancer cells. From 6093 and 6085 nuclei with RNA-seq and ATAC-seq data, 4825 nuclei were identified with both RNA and ATAC profiles (Table 1). CA and transcriptome profiling were also conducted on adult mouse kidney samples, yielding 13 893 RNA-seq profiles and 13 395 sci-ATAC-seq profiles, with 11 296 nuclei exhibiting matched cellular identities. The co-assay of sci-CAR revealed a strong correlation between differential GE and CA in promoter regions, highlighting the dependency of cell-specific GE patterns on CREs. However, the low ATAC coverage in sci-CAR results in excessively sparse sequencing data, which may hinder the identification of cell-specific CREs.

**Table 1:** Data quality of sci-CAR.

Method	Cell type	Number of cells with reads	Data quality (median)	Number of cells with both RNA and ATAC profiles
sci-CAR Data availability: GSE117089	Human-A549-RNA	6093	3809	4825
	Human-A549-ATAC	6085	1456	
	Mice-kidneys-RNA	13,893	1011	11 296
	Mice-kidneys-ATAC	13,395	7987	

RNA: UMIs, ATAC: unique reads

### scCAT-seq

In January 2019, scCAT-seq, based on Smart-seq2 and Tn5 transposase, was introduced as a method to simultaneously profile GE and CA within the same cell [50]. The messenger RNA (mRNA) data generated by scCAT-seq provide full-length transcript coverage and higher resolution for identifying the expression of transcript isoforms beyond the gene level, compared to sci-RNA-seq [51]. Similarly, its CA data achieve sequencing depth comparable to bulk ATAC-seq while capturing chromatin features more accurately than scATAC-seq. CA and GE co-profiling, based on scCAT-seq, was performed for the K562 chronic myelogenous leukaemia cell line, HeLa-S3 cervix adenocarcinoma cell line, HCT116 colorectal carcinoma cell line, a patient-derived xenograft (PDX) model of moderately differentiated squamous cell carcinoma (PDX1), and a PDX model of large-cell lung carcinoma (PDX2), as well as blastocyst and morula-stage cells. The resulting data revealed that TFs act as master regulators of cell fate by binding to CREs, thereby altering chromatin states and driving the formation of cell-specific gene regulatory networks. However, scCAT-seq is only applicable to a limited range of samples. For instance, the sequencing throughput for K562 samples is 192 (Table 2), with only 74 cells simultaneously meeting the quality criteria for both CA and GE profiles (i.e. possessing both CA and GE profiles). Additionally, the throughput for other cell types has not exceeded 500.

**Table 2:** Data quality of scCAT-seq.

Method	Cell type	Data quality (mean)	Number of cells with both RNA and ATAC profiles	Data availability
scCAT-seq	K562-RNA	6.1 × 10^6^	74	NCBI: SRP167062 CNGB: CNP0000213
	K562-ATAC	2.1 × 10^5^		
	HeLa-S3-RNA	2.2 × 10^6^	42	
	HeLa-S3-ATAC	7.7 × 10^4^		
	HCT116-RNA	1.0 × 10^7^	90	
	HCT116-ATAC	3.4 × 10^5^		
	PDX1-RNA	3.9 × 10^6^	176	
	PDX1-ATAC	1.1 × 10^5^		
	PDX2-RNA	4.0 × 10^6^	167	
	PDX2-ATAC	1.1 × 10^5^		
	Blastocyst-RNA	8.5 × 10^6^	43	
	Blastocyst-ATAC	8.8 × 10^4^		
	Morula-RNA	1.3 × 10^7^	29	
	Morula-ATAC	1.1 × 10^5^		

RNA: genome-mapped reads, ATAC: uniquely mapped reads.

### SNARE-seq

In October 2019, the more scalable SNARE-seq technique, based on a micro-droplet platform, was developed for simultaneous single-cell nuclear mRNA and CA profiling [52]. A key innovation of SNARE-seq is the use of Tn5 transposase to capture accessible DNA sequences prior to droplet formation, which prevents the loss of contiguous DNA caused by subsequent high-temperature lysis. This strategy significantly improves the coverage of CA sequences compared to sci-CAR-seq. Initially, SNARE-seq exhibited performance comparable to ATAC-seq, Omni-ATAC, and single-nucleus ATAC-seq for generating CA data of the human GM12878 cell line. Subsequently, SNARE-seq was used to map 1047 coprofiles in human mixed cell cultures; however, it exhibited relatively low mRNA coverage. To improve the performance of SNARE-seq, RNase inhibitor was incorporated to minimize RNA degradation, resulting in approximately a three-fold increase in both UMIs and accessible chromatin peak sites. Following optimization, median yields reached 1159 UMIs and 2264 peaks per nucleus, calculated from 1043 processed cells (Table 3). The landscape of mouse-specific cell types was constructed by generating matched profiles from 5081 neonatal and 10 309 adult mouse cerebral cortex cells. This level of resolution is unattainable by single-omics sequencing approaches. However, variations in mRNA abundance may lead to biased capture of key differentially expressed genes, which may impede the identification of critical genetic differences.

**Table 3:** Data quality of SNARE-seq.

Method	Cell type	Data quality (median)	Number of cells with both RNA and ATAC profiles	Data availability
SNARE-seq	Mixtures of human-RNA	500	1047	NCBI:GSE126074
	Mixtures of human-ATAC	805		
	Mixtures of human-RNA (NP-40-based Nuclei EZ buffer)	1159	1043	
	Mixtures of human-ATAC (NP-40-based Nuclei EZ buffer)	2254		
	Mouse neonatal cerebral cortex-RNA	357	5081	
	Mouse neonatal cerebral cortex-ATAC	2583		
	Adult mouse cerebral cortex-RNA	1332	10 309	
	Adult mouse cerebral cortex-ATAC	2000		

RNA: UMIs, ATAC: accessible sites.

### Paired-seq

**Figure 2:**
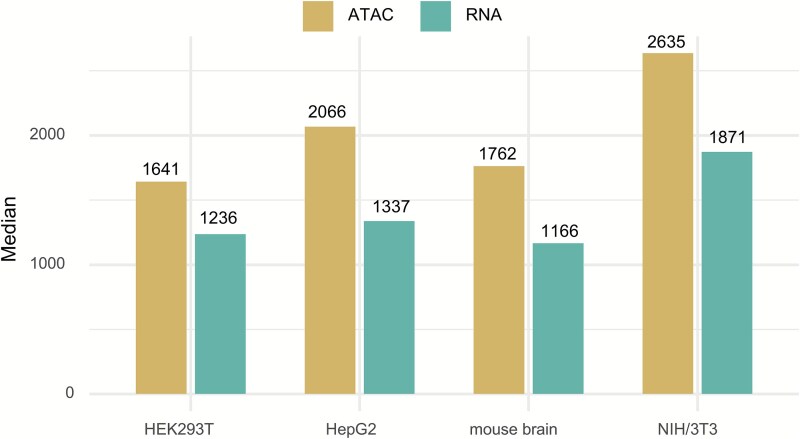
Median uniquely mapped DNA and RNA reads per nucleus for Paired-seq (Data availability: GSE130399).

Paired-seq, published on 6 November 2019, employs iterative rounds of pooling and splitting to assign unique combinations of barcodes to each cell, enabling simultaneous high-throughput sequencing of a cell’s CA and transcriptome [53]. In a mixed sample, Paired-seq showed a low cell barcode recovery rate of 18.9%, which may be attributed to the loss of cell identity information during combinatorial indexing. Of the 6000 profiled cells, 2053 cells with both RNA and ATAC reads were recovered. However, Paired-seq exhibits relatively low and inferior coverage of RNA reads compared to ATAC reads. For instance, sci-CAR achieves a median of 3809 RNA UMIs for A549 cells, whereas Paired-seq achieves a median of only 1337 RNA UMIs for HepG2 cells. In comparison, sci-CAR achieves a median of 1456 ATAC UMIs for A549 cells, whereas Paired-seq achieves a median of only 2066 ATAC UMIs for HepG2 cells (Fig. 2). These results indicate that Paired-seq demonstrates lower RNA sequencing quality compared to sci-CAR. Paired-seq has been used for the high-throughput acquisition of joint RNA and ATAC profiles from 15 191 nuclei out of 30 000 sequenced from adult mouse cerebral cortex, as well as 12 155 nuclei out of 20 000 sequenced from embryonic mouse forebrains at E12.5 and E16.5. Integrated analysis of these data revealed that the promoters of differentially expressed genes exhibit greater dynamics in CA. Although Paired-seq can decipher the gene regulatory programs underlying cell fate at single-cell resolution, the complex multi-round split-and-pool strategy presents challenges for large-scale adoption.

### ASTAR-seq

ASTAR-seq was developed using the Fluidigm C1 microfluidic platform in July 2022. It is a single-cell sequencing technology, similar to scCAT-seq, and enables the simultaneous measurement of the whole transcriptome and CA. Full-length strategies capture entire transcripts, enabling isoform-level analysis, splicing inference, allele-specific expression, and transcript boundary mapping at the cost of lower throughput and higher per-cell cost. By contrast, 3′ capture strategies preferentially sample the 3′ ends with UMIs, providing robust gene-level quantification at higher throughput and lower cost but with limited information on isoforms and splicing. Compared with high-throughput sequencing methods, ASTAR-seq offers superior sequencing depth and enhanced sensitivity in signal detection, making it suitable for analysing samples of incompletely established cell lineages or undergoing organ differentiation. Furthermore, the ability of ASTAR-seq to detect rare transcripts and isoforms across diverse cellular states facilitates a more comprehensive and nuanced investigation of transcriptional regulatory networks. As representative low-throughput single-cell multi-omics sequencing techniques that support whole-transcriptome analysis, both ASTAR-seq and scCAT-seq were used for ATAC and RNA coprofiling of human K562 cells (Table 4). While scCAT-seq generated a higher total read count, ASTAR-seq demonstrated a greater proportion of reads mapping to gene regions and chromatin-accessible peaks, indicating superior sequencing quality and reliability in capturing both transcriptomics and epigenomic features. This comparison underscores the respective strengths of ASTAR-seq and scCAT-seq in multi-omics studies requiring high-resolution and sensitive detection at the single-cell level. The findings suggest that ASTAR-seq enables the deconvolution of the dynamic decision-making trajectories that govern cell fate during the differentiation of primary erythroid progenitor cells, revealing differences in CA and the regulatory mechanisms of TFs at various stages of differentiation.

**Table 4:** Data quality of ASTAR-seq.

Method	Cell type	Number of cells with reads	Data quality (mean)	Number of cells with both RNA and ATAC profiles (passed both QC)
ASTAR-seq (NCBI: GSE113418)	K562-RNA	96	1.2 × 10^6^	291
	K562-ATAC	96	8.8 × 10^4^	
	BJ-RNA	96	1.1 × 10^6^	
	BJ-ATAC	96	7.1 × 10^4^	
	JK1-RNA	96	5.2 × 10^5^	
	JK1-ATAC	96	3.7 × 10^4^	
	Jurkat-RNA	96	6.3 × 10^5^	
	Jurkat-ATAC	96	6.3 × 10^4^	
	Erythroblast-RNA	480	6.0 × 10^5^	273
	Erythroblast-ATAC	480	4.3 × 10^4^	

RNA/ATAC: uniquely mapped reads.

### SHARE-Seq

On 23 October 2020, SHARE-seq was introduced as an advanced single-cell multi-omics technology based on the foundational principles of SPLiT-seq [54] and Paired-seq^55^. SHARE-seq employs three rounds of hybridization blocking to enable highly sensitive and high-throughput coprofiling of RNA expression and CA at single-cell resolution. SHARE-seq exhibits superior stability compared to SCi-CAR, SNARE-seq, and Paired-seq, as evidenced by consistently higher mapping rates, reduced doublet rates, and enhanced species discrimination accuracy in mixed-species experiments (see Table 5 for partial data statistics) [55]. However, similar to other high-throughput single-cell RNA and ATAC co-assay platforms, SHARE-seq produces RNA reads that are primarily enriched in intronic regions due to the 3′ sequencing strategy, yet it retains a robust capacity to investigate cell population heterogeneity at the transcriptional level. To investigate the dominant roles of the CA and transcriptome in cell fate determination during development, coprofiling was performed on 34 774 cells from mouse skin, encompassing highly proliferative, quiescent, and stem cell populations. Owing to the asynchronous changes observed in CA and GE during cell differentiation (as CA changes often precede transcriptional changes), the authors introduced a paired chromatin peak–gene approach to assess cell lineage decisions. The results revealed that genes associated with cell fate determination exhibit a greater number of chromatin peak associations, while cell cycle-related genes exhibit relatively few peak–gene pairs. These findings suggest that changes in chromatin can predict future cell lineage decisions at an earlier stage than changes in RNA expression. In summary, SHARE-seq offers a cost-effective and high-throughput sequencing platform, making it a valuable tool for investigating chromatin potential and lineage choice.

**Table 5:** Data quality of SHARE-Seq.

Method	Cell type	Number of cells for passed QC	Data quality (average)	Data availability
SHARE-Seq	Mixture of human (GM12878) and mouse (NIH/3 T3) -RNA	2244	2545	NCBI: GSE140203
	Mixture of human (GM12878) and mouse (NIH/3 T3)-ATAC		8252	

RNA: UMIs, ATAC: unique fragments.

### ISSAAC-seq

**Figure 3:**
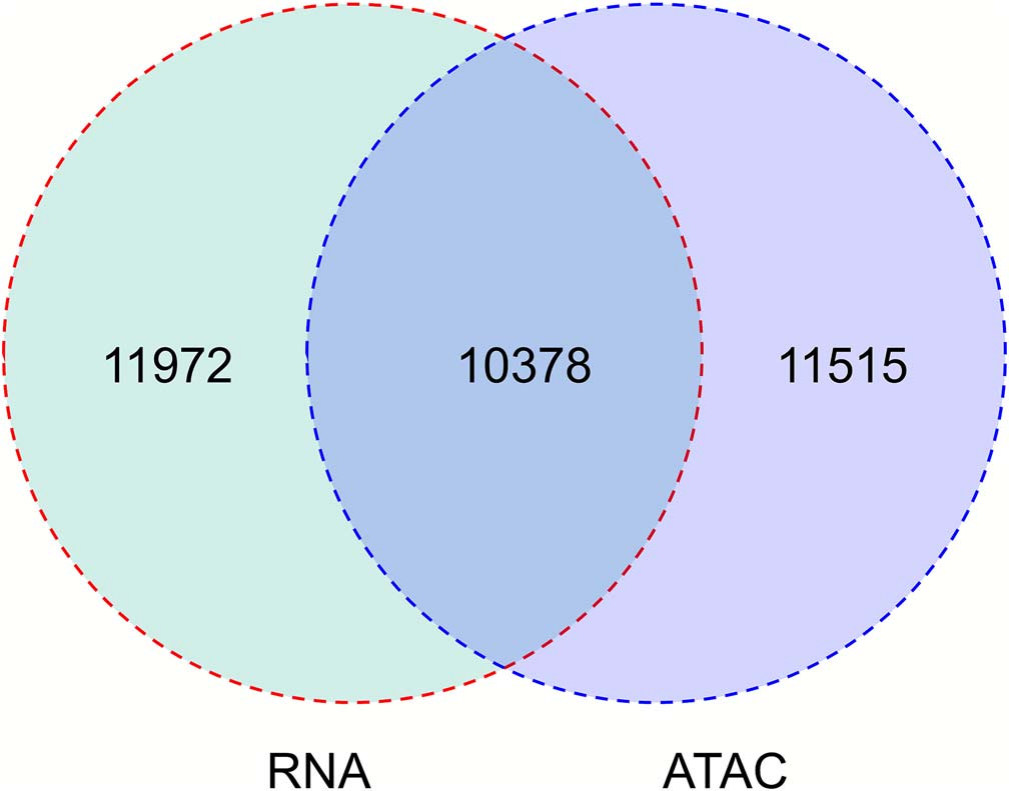
Number of mouse cortex cells analysed by ISSAAC-seq with ATAC and RNA profiles (10,378 cells with both RNA and ATAC profiles; data availability: E-MTAB-11264).

On 20 July 2022, ISSAAC-seq was introduced as a highly sensitive and scalable strategy for decoding rare cell types and cell type-specific CREs based on the joint profiling of CA and GE [56]. Assisted by the sequencing HEteRo RNA–DNA-hYbrid (SHERRY) technique and single-cell ATAC-seq technologies, this method performs DNA tagging of chromatin-accessible regions, mRNA UMI identification, and RT within the nucleus, thereby preventing molecular structural damage to DNA or RNA that may occur during physical separation of chromatin and RNA. Notably, ISSAAC-seq enables flexible library preparation using either flow cytometry or microfluidic droplet systems, depending on the number of sample cells. To optimize sequencing quality, ISSAAC-seq systematically evaluates the impact of various incubation temperatures and chemical reagents on read retention rates. The results demonstrated that RNA RT is most efficient at 30°C and the addition of RiboLock effectively mitigates RNase-mediated RNA degradation. Furthermore, elevated temperatures were found to exacerbate RNA degradation, whereas the ATAC library exhibited greater thermal stability compared to the RNA library. In various cell lines, including the mixed human-mouse cell lines, ISSAAC-seq achieved excellent read detection rates for both modalities, with data quality comparable to that of the 10x Multiome kit. Moreover, its quality and sensitivity surpassed those of other publicly available high-throughput methods, such as SNARE-seq and Paired-seq. Joint RNA and ATAC analysis using ISSAAC-seq in the mouse cerebral cortex revealed that ATAC-seq can further resolve cell type heterogeneity that is not detectable at the transcriptomic level (Fig. 3). Notably, ISSAAC-seq validated the conclusion of SHARE-Seq, demonstrating that chromatin changes can serve as earlier predictors of lineage decisions than RNA changes. Chromatin exhibits a delayed response to decreases in gene transcription, likely due to the slower transition of chromatin states at promoter regions. These findings support a ‘chromatin priming’ model in which regulatory elements gain accessibility and TF occupancy before overt transcriptional changes, thereby encoding lineage bias in advance; conversely, chromatin states can exhibit slower turnover than mRNA, leading to a delayed chromatin response when transcription decreases.

## Data quality assessment of joint profiling by the same criteria

Single-cell joint RNA and ATAC sequencing technologies exhibit distinct sequencing characteristics at different developmental stages. For instance, early methodologies were more adept at resolving transcriptomic landscapes, while subsequent methodologies showed significantly enhanced sensitivity and detection of CA at the single-cell level, albeit often at the expense of RNA data quality. Continual advancements in sequencing platforms and chemistry have gradually stabilized the quality of both RNA and ATAC measurements. Consequently, single-cell joint RNA and ATAC profiling has increasingly become dominated by commercial multi-omics solutions, particularly those developed by 10x Genomics. Currently, scRNA-seq data are typically structured as gene-by-cell matrices for downstream analysis, whereas scATAC-seq data are generally organized in the form of peak-by-cell matrices. The number of expressed genes and CA peaks detected in each cell serve as indicators of data quality across different multi-omics modalities. Therefore, we adopted the evaluation methodology established by SNARE-seq to assess the existing single-cell joint RNA and ATAC sequencing technologies under a unified set of criteria. Specifically, RNA quality was evaluated by the median number of expressed genes across all cells, while CA was assessed by the median number of accessible peaks. This approach enabled a more comprehensive comparison of sequencing quality across different platforms and provided insights into future technological developments.

**Figure 4:**
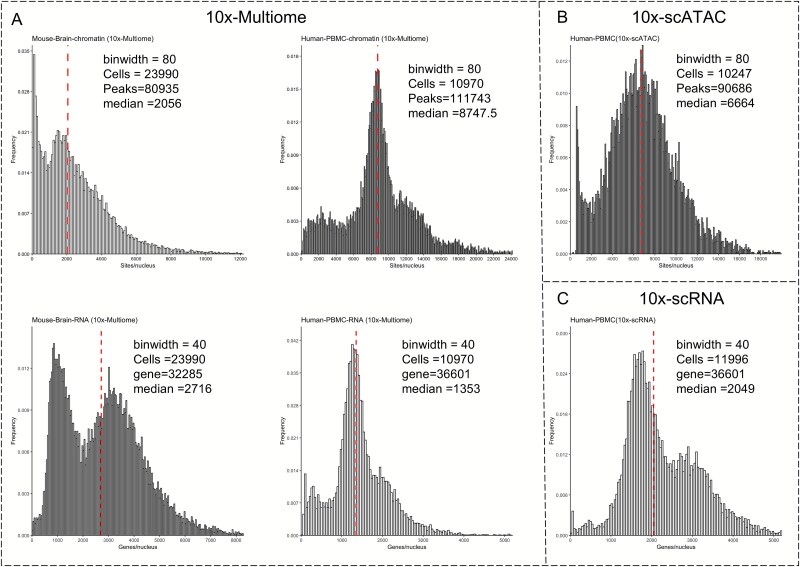
Sequencing quality of 10x Genomics. (A) Evaluation of 10x Multiome GX and CA data from mouse brain and human PBMCs. (B) Evaluation of 10x Genomics scATAC-seq data from PBMCs. (C) Evaluation of 10x Genomics 3′ scRNA-seq data from PBMCs.

**Figure 5:**
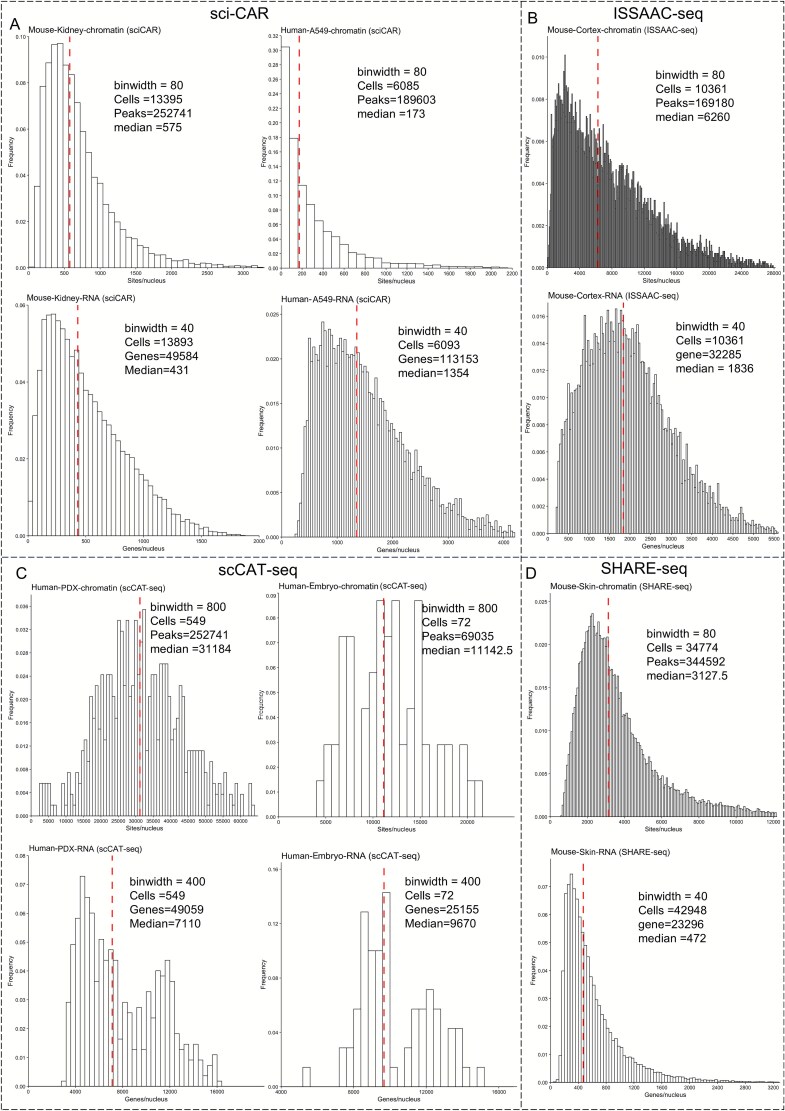
Sequencing quality of joint profiling at different throughput levels. (A) High-throughput evaluation of human A549 cells and mouse kidney using sci-CAR. (B) High-throughput evaluation of mouse cortex using ISSAAC-seq. (C) Low-throughput evaluation of full-length transcriptome in human lung cancer (PXD) using scCAT-seq. (D) High-throughput evaluation of mouse skin using SHARE-seq.

To objectively evaluate quality differences across sequencing technologies, we selected the widely adopted and well-validated 10x Genomics dataset as a reference benchmark, given its commercial availability, broad adoption across diverse tissues and species, standardized chemistries and analysis pipelines, and extensive peer-reviewed evaluations. These factors provide a stable baseline for cross-study comparability and for assessing sensitivity, cell recovery, doublet rate, and cross-modal concordance. Specifically, 10x multi-omics data from mouse brain and human PBMCs were selected as the benchmarks for joint sequencing, while 10x 3′ scRNA-seq and scATAC-seq data from human PBMCs were used as the benchmarks for CA and transcriptomics assays, respectively. As shown in Fig. 4, the single-omics scATAC technology and ATAC-seq of joint sequencing exhibit high concordance in human PBMCs. At comparable cell throughput, the median number of peaks detected by scATAC and 10x multi-omics ATAC accounted for 7.3% (6664/90 686) and 7.8% (8747.5/111 743) of the total peaks (Fig. 4A and B), respectively, despite the increased background noise in joint sequencing, which can lead to a higher number of detected peaks. Conversely, the transcriptomics data showed a greater variation, with the single-omics scRNA-seq exhibiting a higher median number of detected genes compared to the 10x multi-omics RNA, despite similar total gene counts. This difference may be attributed to the relatively milder experimental conditions typically used during Multiome preparation, which preserve nuclear integrity but can result in incomplete mRNA release. As early adopters, we found that the peak detection rate of sci-CAR in human A549 cell lines was suboptimal, with a median of only 173 peaks per cell. Although the median gene count for RNA was comparable to that of the 10x Multiome platform, the signal-to-noise ratio remained low, even though the total number of detected genes reached 113,153 (Fig. 5A). Similarly, the sensitivity of the chromatin reads in scCAT-seq was found to be low, suggesting the need for further improvement. However, scCAT-seq demonstrated a high number of detection features (peaks/genes) in both human embryonic cells and PXD cells, with median transcriptomics feature counts reaching 7110 and 9670, respectively (Fig. 5C). This is largely attributed to its full-length transcriptome sequencing, which enables the detection of transcripts and isoforms, providing higher-resolution features.

**Figure 6:**
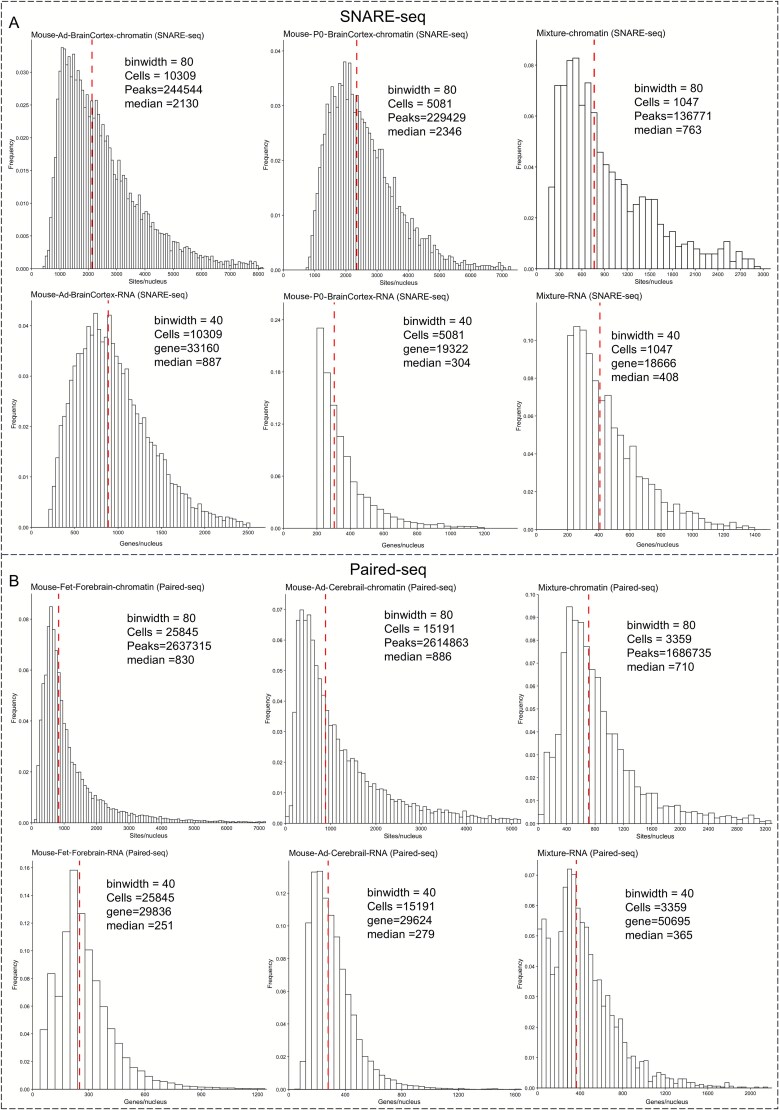
Various joint profiling sequencing quality in mice. (A) Evaluation of SNARE-seq in adult mouse brain cortex, P0 brain cortex, and mouse–human mixed samples. (B) Evaluation of Paired-seq in mouse foetal forebrain, adult cerebral cortex, and mouse–human mixed samples.

Considering that the mouse genome shares ~85% homology with the human genome, mice are widely used as biological models to simulate various disease scenarios to better understand the underlying mechanisms. Consequently, multiple single-cell joint profiling approaches, combining RNA and ATAC sequencing, have been applied in mouse models for integrated analysis. For the 10x multi-omics data, the median gene count in the mouse brain is 2716, which is higher than that in humans, while the peak count is much lower, with only 2056 detected peaks. The median peak count of sci-CAR in kidney cells is higher than that in the A549 cells, while the median gene count is only 431, highlighting the instability of the sequencing performance across species. The latest ISSAAC-seq technology shows a median gene count of 1836 in mouse cerebral cortex cells, which is slightly lower than that of the 10x Multiome RNA but achieves a median peak count of 2056, surpassing the 10x Multiome ATAC (Fig. 5B). SHARE-seq also demonstrates high sensitivity in peak detection, with a median peak count of 3127.5, being the highest among the tested sequencing technologies; however, its RNA performance is relatively low (Fig. 5D). Both SNARE-seq and Paired-seq have been applied to mouse tissues at different developmental stages (Fig. 6). Paired-seq demonstrated stable performance for both RNA and ATAC across varying cell throughputs. Compared to SNARE-seq, Paired-seq detected a greater total number of peaks but showed relatively lower median peak count per cell (Fig. 6B). In contrast, SNARE-seq exhibited instability in the RNA quality across different tissues, with a median RNA count of 887 in the adult brain cortex and only 304 in the P0 brain cortex (Fig. 6A). Overall, balancing the sequencing quality between different omics layers is a major challenge for joint ATAC and RNA sequencing technologies.

### Analytical tools and applications of joint profiling

#### The balance between sequencing costs and sequencing sensitivity

Joint single-cell CA and transcriptome sequencing technologies enable simultaneous mapping of the gene and CRE profiles within the same cells. However, balancing the cost of these technologies with the quality of the data remains a challenge in practical applications. ASTAR-seq and scCAT-seq focus on low-throughput yet high-coverage profiling, are sensitive to sequencing quality, and enable high-resolution analysis of the dynamic interplay between gene transcription and chromatin states. Both methods have an average sequencing cost of ~$0.30 per cell. ASTAR-seq, based on the Fluidigm platform, incurs lower overall costs and delivers higher sequencing quality compared to scCAT-seq, which utilizes a microfluidic platform. This makes ASTAR-seq particularly well suited for detailed investigations of transcript isoforms. In contrast, high-throughput methods such as Paired-seq (~ $0.05 per cell) and SHARE-seq (~ $0.0043 per cell) offer cost-effective joint profiling of transcriptomes and CA. Due to their relatively low sensitivity, they are most appropriate for samples with pronounced cellular heterogeneity. sci-CAR, with an estimated cost of $0.30 per cell, excels in the analysis of transcriptome heterogeneity, whereas SNARE-seq, costing ~$0.20 per cell, is particularly proficient in identifying heterogeneity in CA. For tasks requiring high sensitivity across both transcriptomic and CA signals, ISSAAC-seq (~$0.20 per cell) represents a strong alternative. However, as a self-implemented method, it requires adherence to published protocols and the preparation of numerous biochemical reagents and laboratory instruments, rendering it impractical for nonspecialists. Conversely, when cost is not a limiting factor, 10x Multiome (~$0.40 per cell) provides an optimal solution as a commercially available platform with mature reagent kits. It has been successfully applied across multiple biological tissues and does not require users to optimize experimental conditions or prepare large quantities of custom reagents, thereby reducing experimental uncertainty and lowering technical barriers.

#### Analytical tools and workflow for single-cell transcriptomics

**Figure 7:**
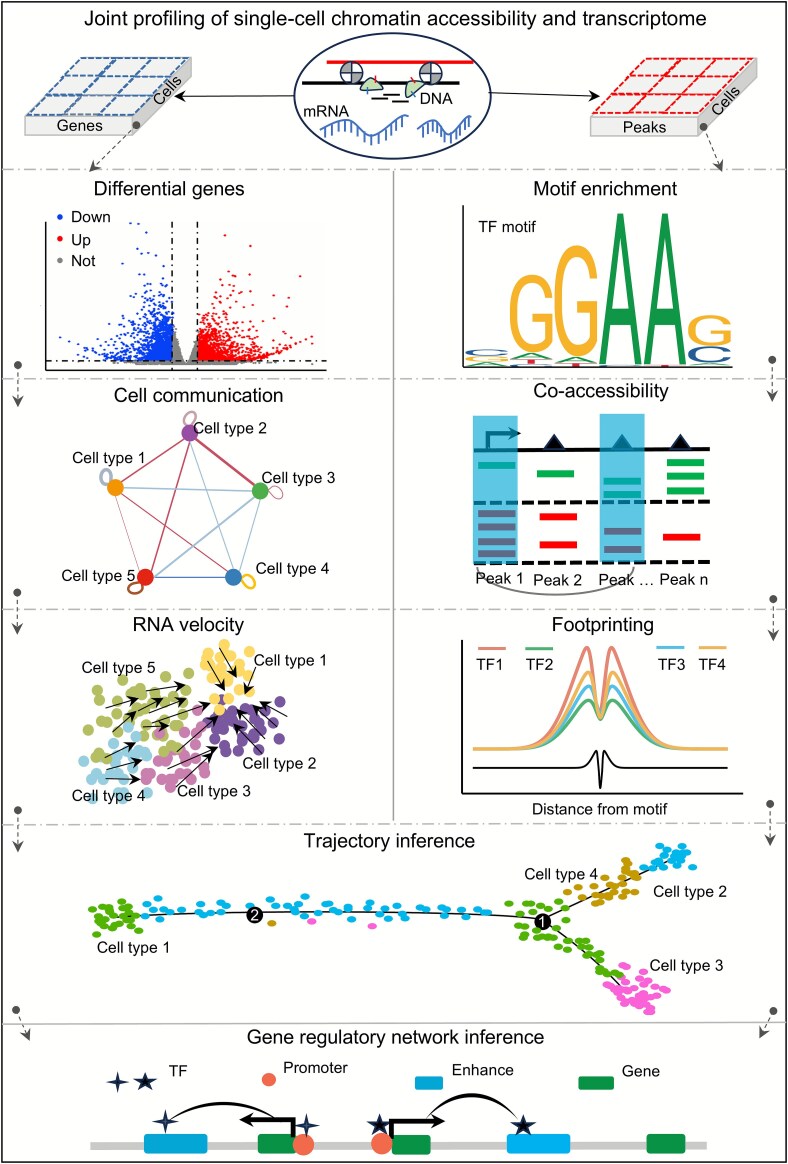
Analytical tools for single-cell joint profiling. Differential genes, cell communication, and RNA velocity are applicable to scRNA-seq analyses (left) and motif enrichment, co-accessibility, and footprinting are suitable for scATAC-seq analyses (right). Trajectory inference can be used for both scRNA-seq and scATAC-seq, while gene regulatory network inference (bottom) is applicable to single-cell joint profiling of CA and transcriptome.

Consequently, various single-cell computational analysis tools [57–60] can be applied to study intercellular heterogeneity (Fig. 7). Single-cell transcriptomic data are the most widely applied and have the largest accumulation, thereby involving a wide range of computational analysis tools. Differential gene analysis is a common and fundamental tool in transcriptomics [61] and is often performed using Scanpy (developed in Python) [62] and Seurat (developed in R) [63]. At single-cell resolution, differential gene analysis is often used to determine upregulated and downregulated genes between different cell types, in order to explore the biological functions and identities of cells required for downstream analyses. After cellular identities are established, cell–cell communication networks can be constructed based on ligand–receptor interactions, enabling the exploration of potential intercellular signalling patterns [64]. Similarly, identification of ‘hub’ signalling molecules within the microenvironment can provide valuable insights into drug development and targeted therapies. Additionally, cellular developmental trajectories can map the dynamic processes of cell development, thereby identifying key cell fate decision points. RNA velocity calculates the rate of GE changes and potential developmental directions based on the ratio of unspliced and spliced mRNA expression levels in cells, thus enabling the reconstruction of cellular developmental trajectories [65]. Not limited to RNA velocity, Monocle2 can also infer cellular developmental trajectories based on differential gene analysis [66].

The canonical and indispensable workflow in single-cell transcriptomics is cell type identification, encompassing quality control, data normalization, feature selection, data scaling, dimensionality reduction, clustering, and differential gene analysis. For datasets with a small number of cells or scenarios requiring multimodal integration, Seurat offers sufficient functionality (https://satijalab.org/seurat/articles/pbmc3k_tutorial) [63]. In contrast, Scanpy demonstrates superior scalability for large datasets and is particularly well suited for downstream tasks such as machine learning model construction (https://scanpy.readthedocs.io/en/latest/tutorials/basics/clustering-2017.html) [62]. When downstream analytical objectives involve elucidating molecular signalling pathways or identifying potential drug targets, cell–cell communication can be constructed using CellChat or CellphoneDB. CellphoneDB, implemented in Python, is suited for integration with Scanpy (https://github.com/ventolab/CellphoneDB) [67], whereas CellChat, implemented in R, is optimized for use with Seurat (https://github.com/sqjin/CellChat) [64]. If the research focus is on identifying developmental nodes and cell fate, Monocle, developed in R, is well suited for integration with Seurat (https://cole-trapnell-lab.github.io/monocle-release/), while scVelo, developed in Python, aligns well with Scanpy (https://velocyto.org/velocyto.py/tutorial/index.html), although it requires additional BAM files as input.

#### Analytical tools and workflow for single-cell CA

Single-cell CA data are not only highly sparse but also exhibit a wide diversity of data features. After sequencing, raw reads are processed into a fragments.tsv.gz file containing genomic coordinates, which serves as the basis for downstream analysis by users or computational tools. However, for many computational analyses [57, 68, 69], it is still necessary to convert the fragments file into a cell-feature matrix, similar to those used in transcriptomics studies, to facilitate further processing. Cell-tile, cell-gene score, and cell-peak are commonly used data representations for CA. The cell-tile approach divides the genome into fixed-length tiles and counts the CA of each tile in individual cells; however, this method tends to be overly sparse. The cell-gene score approach evaluates gene activity based on the distribution of accessible fragments near genes, thereby providing a representation of gene activity that is analogous to transcriptomics data; however, it neglects the influence of distal CREs on gene activity. In contrast, the cell-peak method performs peak calling on accessible fragments from all cells and subsequently quantifies the accessibility within each peak region for every cell. Consequently, the cell-peak method has become the predominant representation in recent studies. Peaks, as potential regions of various CREs, frequently harbour binding sites for multiple TFs. By analysing the enrichment of TF-binding motifs within these regions, it is possible to infer which TFs are likely to bind at these sites. This approach facilitates the elucidation of the regulatory mechanisms through which TFs modulate GE. CREs regulate GE in a coordinated manner through both distal and proximal chromatin looping interactions. The co-accessibility established by these chromatin loops provides a mechanistic framework for understanding how chromatin state facilitates the precise control of gene regulatory processes [70]. TF motif enrichment analysis may identify binding motifs for multiple TFs within the same peak region [71]; however, due to the competitive nature of TF binding, typically only a few specific TFs are actually bound at each peak. The TF-bound regions are protected from cleavage by transposases like Tn5. TF footprinting analysis uses this “protective effect” by detecting the regions with reduced transposase accessibility, thereby inferring the precise TF-binding sites and actual occupancy of individual TFs [72, 73].

The core workflow for single-cell CA analysis largely parallels that of single-cell transcriptomics, with the primary distinction being the format of the input data. When the input is a peak-by-cell matrix, the Signac package is well suited for executing the workflow and performing downstream analyses (https://stuartlab.org/signac/articles/pbmc_vignette). If the input is a fragment file, both Signac and ArchR can process the data to perform analyses such as TF footprinting, trajectory inference, co-accessibility mapping, and motif enrichment; however, peak calling must first be conducted on the fragment file. ArchR provides methods for pairing nonjoint profiling scATAC-seq and scRNA-seq datasets, enabling joint single-cell multi-omics analysis across different batches and platforms (https://www.archrproject.com/articles/Articles/tutorial.html), although it has not been actively maintained or updated in recent years. In contrast, Signac offers comprehensive tutorials for working with 10x Multiome and SNARE-seq data, making it a more suitable choice for joint profiling applications.

### Workflow and applications of joint profiling

The advantage of joint sequencing technology lies in its ability to simultaneously obtain transcriptomic and CA information at single-cell resolution, thereby establishing precise links between genes and peaks and uncovering coordinated regulatory mechanisms among multiple molecules during cell development. Signac provides a basic workflow for joint profiling analysis, including quality control, transcriptomic data processing, CA data processing, cell annotation, visualization, and gene–peak association analysis (https://stuartlab.org/signac/articles/pbmc_multiomic). Within the same analytical framework, it retains ATAC-specific features such as gene activity and motif analysis, thereby supporting multimodal joint learning and downstream analyses. In downstream analyses of joint profiling, one key contribution is the precise construction of gene regulatory networks within specific cellular subpopulations—an achievement that single-modality transcriptomics or CA profiling alone cannot accomplish. By identifying key regulatory hubs and elucidating the mechanisms of CREs and TFs for target genes, researchers can improve the accuracy of cell lineage mapping and enhance the detection of cellular heterogeneity within disease microenvironments [74].

SCENIC+ is a specialized bioinformatics tool for constructing single-cell regulatory networks based on single-cell transcriptomics and CA [74]. Developed in Python, it requires single-cell transcriptomic data to be processed using Scanpy, and CA data to be processed using pycisTopic [75]. Subsequently, regulatory network construction can be completed using the automated SnakeMake scripts provided by SCENIC+ (https://scenicplus.readthedocs.io/en/latest/tutorials.html). For researchers with an R language background, our previous publication [76] provided a data format conversion script for transcriptomic results processed with Seurat and CA results processed with ArchR, serving as a reference for regulatory network construction (https://github.com/Hongfeipower/HTN). Notably, joint profiling is not only limited to animal tissue samples but can also be used for reconstructing regulatory networks in plants. However, in plant applications, nuclei are the primary input for joint sequencing library preparation; nuclei are obtained from frozen tissues by gentle chopping, on-ice shaking, low-speed centrifugation, separation, collection, and washing. *Arabidopsis thaliana*, a model organism for several economically important crops, has evolved a variety of specialized cell and tissue structures to adapt to harsh environmental stresses, such as drought, high salinity, and low temperatures. Reconstructing its gene regulatory networks can help elucidate the molecular mechanisms by which differential GE mediates plant phenotypic and physiological responses to such environmental stresses. For example, 10x Chromium single-cell multi-omics technology has been applied to *Arabidopsis* root tissues under osmotic stress to construct GE regulatory networks [77]. The results revealed that changes in CA can predict future cell differentiation trends earlier than changes in GE and that cell-specific alterations in gene regulatory networks are primarily driven by TFs and CREs. These insights provide valuable guidance for crop improvement.

## Discussion

Joint profiling of single-cell CA and transcriptome has enabled the mapping and characterization of numerous cell-type-specific regulatory networks, particularly advancing our understanding of cell fate decisions and the heterogeneity of disease microenvironments. Nevertheless, achieving a balance in data quality between the transcriptome and CA layers remains a significant challenge, as it is difficult to attain single-omics-level data quality in both modalities simultaneously. In addition, most high-throughput joint profiling approaches rely on 3′ RNA sequencing, leaving a gap in 5′ transcriptome data. Notably, the integration of 5' RNA sequencing with CA analysis would be invaluable for elucidating causal relationships between chromatin states and transcript isoform variations. Although long-read RNA-sequencing technologies—such as Pacific Biosciences and Oxford Nanopore Technologies [78, 79]—can potentially fill this gap by providing full-length transcript information, improvements in sequencing throughput and cost are still required for their broader application in joint profiling.

The rational application of computational tools is essential for the biological interpretation of single-cell data. Beyond mathematics-based tools for functional analysis in single-cell biology, a variety of deep learning–based tools [80] for single-cell multi-omics pairing, translation, and integration have also emerged; for example, scButterfly achieves consecutive translation from the epigenome to the transcriptome and then to the proteome [81]. These tools facilitate the restoration of omics signals in joint profiling technologies and enable accurate cell-to-cell matching for unpaired multi-omics data. However, the development of computational methods for analysing full-length single-cell transcriptomes, including alternative splicing, transcript variants, and isoforms, remains insufficient. Additionally, integrating CA data from different batches or sequencing platforms poses significant challenges. When peaks are merged based on total sample fragments, unique peaks that are specific to individual sub-samples may be lost. Moreover, the original peak matrices of subsamples often differ in chromatin intervals and feature numbers, which complicate the integration process. Therefore, the development of new feature descriptors for CA, such as utilizing TF expression levels and fragment enrichment scores, is an important direction for future research.

Current sequencing technologies are capable of simultaneously measuring multiple omics signals, such as DNA methylation, transcriptome, and CA, within a single cell. This integration introduces the DNA methylation perspective into regulatory network analysis, enabling a comprehensive understanding of the dynamic interplay between epigenetics and transcriptional regulation. The incorporation of chromatin interaction and spatial omics approaches into joint profiling of single-cell CA and transcriptome shows excellent potential for constructing more accurate and detailed multidimensional gene regulatory network maps, both within and between cells.

**Key Points**

- Different platforms of joint profiling of single-cell CA and transcriptome vary significantly in sequencing quality, throughput, and sample applicability.
- Joint profiling of single-cell CA and transcriptome integrated with bioinformatics tools enables cell type identification and regulatory mechanism elucidation.
- Challenges remain in simultaneously improving RNA and ATAC quality and achieving cross-platform data integration.

## Data Availability

Not applicable.
